# Cases Illustrating Symptoms Referable to the Ends of the Extremities

**Published:** 1908-11-07

**Authors:** Thos. D. Savill

**Affiliations:** delivered at the West End Hospital for Nervous Diseases, Welbeck Street, W.


### Hospital Clinics.

### CASES ILLUSTRATING SYMPTOMS REFERABLE TO THE ENDS OF THE EXTREMITIES.

Epitome of a Clinical Lecture by THOS. D. SAVILL, M.D.Lond., F.R.C.P., delivered at the West End Hospital for Nervous Diseases, Welbeck Street, W.

Gentlemen,—I happen to have a series of cases illustrating symptoms which are referable to the ends of the extremities, and I have thought that it might not be uninteresting to collect them together and demonstrate them to you this afternoon.

In the first place, I would like to remind you of the fact that the ends of the extremities, that is to say, the hands and the feet, present two peculiarities which they share, apart altogether from the fact that they are both exposed to very much the same local conditions. Thus both the hands are exposed to the air and to the action of water and to occupations, and all these may result in eruptions which are more or less symmetrical. But with these we are not concerned now so much as with the two special physiological peculiarities to which I want to draw your attention. I would ask you to remember that the ends of the extremities have these two peculiarities in common. They each have longer neurons than other parts of the body, and consequently they show a special tendency to trophic changes. The second peculiarity is that in each there is what might be called a "dead end" to the circulation, a kind of backwater where the blood stream is sluggish. This is a purely mechanical factor, but it is an important one, and one which is very often overlooked. It is specially important in toxic states, for the toxins are here delayed. Bearing these common peculiarities in mind, we see that there are two groups of cases which correspond to them.

First, we meet with nerve cases, such as lesions of the lower neuron. This patient here, a man of 60 years of age, is a well-marked example of chronic anterior poliomyelitis, progressive muscular atrophy as it is sometimes called. His story is that five or six years ago he first began to notice that his hands were wasting. Now, as you can see, he has marked wasting of the small muscles of the hands. The interossei and the thenar and hypothenar muscles are chiefly affected, and he is already beginning to get his forearms involved. By and by, the muscles of the shoulder girdle will be attacked, and later on the lateral columns of the spinal cord. At present the main lesion is one of the anterior motor cells, and you have a fairly perfect type of a lower neuron paralysis, of which the essentials are flaccid paralysis, muscular wasting, and electrical changes, without alteration of the deep reflexes. These are the three characteristics of a lower neuron lesion as against a lesion of an upper neuron in which you get stiffness, increased deep reflexes, no wasting, except from disuse, and no electrical changes.

There is another nerve disease in which the sensory neurons are affected as well as the motor. This is *syringomyelia*, and in these sections of the cord the changes which occur are well shown. You will notice that there is an increase in the size of the central canal; there is cavitation in fact, with an overgrowth of gliomatous tissue. This is the essential thing in this disease, a progressive gliomatous cavitation. Whether it is congenital or not is a point upon which pathologists are not as yet agreed. Now in such a case we get four sets of symptoms. First we meet with sensory changes, and among others there is that characteristic feature first pointed out by Charcot, loss of thermal sensation. The probable explanation of this is that the thermal tracts lie close to the central canal, and are early involved in the gliomatous overgrowth. Then the anterior horns are involved and muscular wasting results; subsequently the posterior horns and ganglia, causing sensory and trophic changes. In one patient these last were very marked; he shed portions of the skin of his hands like casts; they seemed to slip off like a glove. Finally, fourthly, there is pressure on the lateral columns, which gives rise to spasticity. This patient, a woman, came up complaining of pain in the right side of her head and numbness of her right arm. On examining her, we found there was slight wasting of the thenar and hypothenar muscles, and as you see there is marked thermal anæsthesia, for she is not able, with her eyes shut, to tell the difference between a hot or cold test-tube placed on the skin of her arm. She also presents another feature of interest in that she has some paralysis of her cervical sympathetic. The signs of paralysis of the cervical sympathetic are: first, contraction of the pupil on the affected side; second, narrowing of the palpebral fissure on the same side; and, third, recession of the eyeball or enophthalmos. This patient has not got the pupil sign, but she shows the palpebral and the eyeball sign, and in addition she has another sign—an absence of sweating, or anidrosis, on the affected side. In cases of irritation of the cervical sympathetic there is the converse of this, that is unilateral sweating. This case, I may mention, was at first regarded as a pure sympathetic lesion, but it is not uncommon to find the sympathetic affected in cases of syringomyelia. The symptoms appear to be due to the involvement of the pupil dilator fibres within the cord. These, then, are two cases of lower neuron lesions. In both the extremities are first affected, but there is this distinction, that in the first the symptoms are mainly motor, while in the second they are mainly sensory.

We now turn to a different class of case. There are quite a number of them—eight in fact—and they are all due mainly to faults in the circulation or vaso-motor apparatus, coupled sometimes with **toxæmia.**

(1) The first is the classical condition known as Raynaud's disease, in which the changes in the skin may lead to ultimate gangrene. (2) Secondly, we met with a number of conditions which may all be classed as being due to general feebleness of the circulation—cold "dead" hands, blue hands, cold feet, and such like conditions which are so common that I need not show them to you this afternoon. (3) Thirdly, we have a condition which was called erythromelalgia by Weir Mitchell. This is a painful red swelling of the hands and feet, and it is nearly always symmetrical. This patient is an example of such a condition. She is a case of erythromelalgia which I saw and described in 1901,* when she came to me complaining of pain, swelling, and redness in her hands dating from 1897. A point in her case was that her distress got very much worse when she went to bed or even when she lay down on the sofa. In fact, whenever she assumed a horizontal position pain and distress came on. The treatment that did her most good was galvanism, the constant current frequently applied. She was also given calcium chloride, and got on quite well. At present she has some red patches on her feet, and some time ago she suffered from what her doctor held to be erythema nodosum of rheumatic origin. These red patches on her feet appear to be something of the same sort, and this, then, is a condition which one may describe as a toxo-angeio-neurotic lesion.

A fourth condition is hyperidrosis, excessive sweating of the hands and feet. One bad case was treated successfully with ergot. This girl here complains of such a condition. She also suffers from what she calls blisters, there are crops of (5) herpetic patches on her hands. These vesicles are certainly trophic, and herpes is now generally regarded as being due to a lesion of the posterior root ganglia. There are two practical points as to the local treatment in such cases. You may use either salicylic acid and oxide of zinc, or a weak solution of nitrate of silver in spiritus ætheris nitrosi. The latter darkens the skin a little, but it also hardens it, and both are valuable as local applications. Internal treatment is also necessary.

The sixth lesion is a trophic condition of the nails. This woman is forty-three years of age, and came with a vesicular eruption on her hands which recurred from time to time since she was thirteen years old. You will notice there are sago-like granulations under the epidermis on the palm of her hands, and associated with this vesicular condition she has a curious condition of her nails which dates from nine years ago. They are curved, concave instead of convex, thin and turned up at the ends, with a certain amount of hyperkeratosis underneath them. The condition is a trophic one. (7) The next case again is a very old patient of mine, who first came because her face became swollen and she got swelling of her hands due to urticaria. Now, after eight years, she comes complaining of such little sago-like granulations on the palms of her hands. This eruption is associated with swelling and flushing of the face, and she says that she feels very tired. Now in all probability this means that she has a sort of chronic toxæmia. Her lesion is mainly a toxic one.

(8) Finally, there is one more lesion, hyperkeratosis palmaris. This case which I show to you is a very interesting one. In a great many cases hyperkeratosis is an acquired condition, but in a few it is congenital, as it happens to be in this case. This woman is twenty-nine years of age, and is one of a family of eight. Three of her generation are afflicted with a similar condition of the palms of the hands and soles of the feet; the hyperkeratosis is not found elsewhere on the body, but she has a general dryness of her skin. She is being treated with tar and salicylic acid, 10 gr. to the ounce.

In conclusion it appears to me that there are always three factors to be remembered in considering cases like these you have seen. Firstly, there is the sluggishness of the circulation. This is a merely mechanical factor; you have to deal with a loop, where the blood current is slowed and where there is partial stasis. Such stasis leads to lymph stasis, cell œdema, and finally definite changes in keratinisation of the skin. Then in the second place you have nearly always, at any rate in acquired conditions, some toxic blood condition, and owing to the sluggish circulation at the peripheral ends of the extremities the toxin remains longer and does more damage in these parts. Thirdly, these cases have an inherent weakness of their vaso-motor and trophic centres. Such conditions tend to occur more frequently in some people than in others, and then tend to recur. In nearly all one is able to trace these three factors in varying proportion.

* *The Lancet.*

